# Naturalistic language assessment and brain-network biomarkers in post-stroke aphasia for precision neurorehabilitation

**DOI:** 10.3389/fnhum.2026.1966552

**Published:** 2026-10-15

**Authors:** Jiayi Xia, Song Pei, Song Zhou, Yulong Bai

**Affiliations:** 1 Clinical Research Center, Shanghai Second Rehabilitation Hospital, Shanghai University of Traditional Chinese Medicine, Shanghai, China; 2 Department of Rehabilitation Medicine, Renhe Hospital, Baoshan District, Shanghai, China; 3 Department of Rehabilitation Medicine, Huashan Hospital, Fudan University, Shanghai, China

**Keywords:** brain-network biomarkers, connected speech, ecological validity, naturalistic language assessment, neuropsychological profiling, portable neuroimaging, post-stroke aphasia, precision neurorehabilitation

## Abstract

Advances in naturalistic language assessment, portable neuroimaging, electrophysiology, and brain-network modeling are creating new opportunities to evaluate post-stroke aphasia under conditions closer to real-world communication. Conventional aphasia batteries and isolated linguistic tasks remain essential for diagnosis, severity classification, and treatment planning, but they provide limited information about how language-network activity supports communication in dynamic, naturalistic contexts. This integrative review, informed by a structured literature search and descriptive thematic mapping, synthesizes evidence on connected speech, discourse production, narrative comprehension, interactive communication, and contextual reading after stroke. We examine how multilevel features from these behaviors can be organized into graded, interacting dimensions of lexical-semantic access, phonological encoding, grammatical organization, discourse processing, executive-predictive control, and speech-motor output. We further review multimodal evidence from fNIRS, fMRI, EEG/ERP, lesion-symptom mapping, structural connectivity, and functional connectivity, highlighting residual left-hemisphere language networks, conditional right-hemisphere support, bilateral reorganization, domain-general control systems, and longitudinal recovery. Portable EEG and fNIRS are considered complementary approaches for repeated assessment during less constrained language tasks, with distinct temporal, spatial, and practical trade-offs. Building on this evidence, we propose an operational ecological biomarker framework that links task choice, multilevel feature extraction, multivariate behavioral profiles, neural measures, prespecified clinical questions, and longitudinal outcomes. This framework may support recovery prediction, rehabilitation stratification, neuromodulation target selection, individualized therapy planning, and monitoring of functional communication.

## 1 Introduction: why post-stroke aphasia assessment needs an ecological turn

Instruments such as the Western Aphasia Battery–Revised (WAB) (Kertesz, 2006), the Boston Diagnostic Aphasia Examination (3rd ed.) (BDAE) (Goodglass et al., 2001), naming tests, repetition tasks, comprehension subtests, and reading or writing measures remain indispensable for diagnosis, syndrome classification, severity grading, and treatment planning (Sheppard and Sebastian, 2021; Halai et al., 2022; Kim and Yang, 2022; Castro et al., 2023). These tools provide a shared clinical language and allow clinicians to quantify impairment across core linguistic domains. They are also essential for documenting recovery, determining eligibility for rehabilitation, and comparing outcomes across studies. However, their clinical utility does not eliminate a fundamental limitation: most conventional assessments capture language ability under structured and decontextualized conditions, whereas everyday communication unfolds in dynamic, interactive, and context-dependent situations.

This gap between impairment-based testing and real-world communication is not unique to aphasia. It reflects a broader challenge in neuropsychological assessment: the ecological validity of a test depends on whether it predicts or represents the everyday function it is intended to measure. Chaytor and Schmitter-Edgecombe argued that neuropsychological tests often show only moderate ecological validity when predicting everyday cognitive functioning and that stronger associations are observed when the test and outcome measure assess closely corresponding domains (Chaytor and Schmitter-Edgecombe, 2003). Applied to post-stroke aphasia, this principle suggests that naming, repetition, and sentence-level measures should be judged partly by how well they relate to discourse participation, conversational success, and communicative independence. If the target outcome is real-world communication, assessment must include tasks that approximate the cognitive-linguistic demands of real-world communication.

Constrained aphasia tasks and naturalistic tasks are not separated by a simple presence-versus-absence of lexical, semantic, syntactic, memory, or executive demands. Even a sentence-comprehension item can recruit several of these processes at once. The distinction is instead one of task constraint and observability: naturalistic samples allow multiple linguistic and cognitive processes to vary and interact within the same extended communicative episode. Their value is therefore not that they uniquely contain processes absent from conventional testing, but that they permit simultaneous measurement of several sources of difficulty under conditions that more closely resemble everyday communication.

This ecological gap has become increasingly visible in discourse and connected speech research. Spoken discourse has been described as a clinically meaningful and naturalistic language outcome because it captures linguistic performance at the level of extended communication rather than isolated test items (Stark et al., 2021b). Surveys and reporting-guideline studies further indicate that clinicians and researchers recognize discourse analysis as relevant to aphasia assessment, while also acknowledging major variability in elicitation, transcription, scoring, and interpretation (Stark et al., 2021b). Connected speech and narrative measures can reveal lexical diversity, informativeness, coherence, fluency, syntactic complexity, and communicative efficiency, all of which are more closely aligned with functional communication than single-word measures alone (Wilmskoetter et al., 2019; Alyahya et al., 2020; Schumacher et al., 2020; Ding et al., 2023). These findings suggest that naturalistic language behaviors may provide a more ecologically valid window into the lived communication difficulties of people with aphasia.

At the same time, naturalistic language measures should not be viewed simply as longer or more complex behavioral outcomes. Their deeper value lies in their potential to reveal neuropsychological phenotypes that reflect individual differences in language impairment and recovery. A phenotype in this context refers to an observable behavioral pattern that reflects the interaction between cognitive-linguistic operations and underlying brain-network organization. Connected speech may reflect lexical retrieval and speech-motor output; narrative discourse may reflect semantic integration, temporal organization, and discourse planning; conversation may reflect executive-linguistic control, turn-taking, pragmatic adaptation, and error monitoring. Studies linking discourse production to lesion location, white matter damage, and structural or functional network features support the idea that naturalistic language behaviors can be mapped onto neural mechanisms rather than treated as purely descriptive outcomes (Gleichgerrcht et al., 2016; Wilmskoetter et al., 2019; Schumacher et al., 2020; Ding et al., 2023; Casilio et al., 2025).

Building on this evidence, the present review focuses on how features derived from less constrained language samples can be organized into interacting behavioral dimensions, related to neural measures, and evaluated against prognosis, treatment response, and functional communication outcomes. This behavioral perspective leads directly to the network-level account developed below.

This reconceptualization is timely because the neurobiology of post-stroke aphasia recovery is increasingly understood as a network-level process. Functional imaging and lesion studies indicate that recovery depends on residual left-hemisphere language networks, right-hemisphere recruitment, bilateral reorganization, white matter connectivity, and domain-general control systems (Saur et al., 2006; Geranmayeh et al., 2017; Griffis et al., 2017; Wilson and Schneck, 2021). However, many neuroimaging studies still rely on constrained tasks such as naming, semantic judgment, or sentence comprehension. These tasks are experimentally controlled but may underrepresent the complexity of communication. Naturalistic paradigms, including connected speech, story comprehension, unscripted discourse, and natural speech listening, may therefore help bridge behavioral neuropsychology and brain-network biomarkers.

Several methodological advances have made this ecological turn increasingly feasible. Functional MRI has provided important insights into discourse processing and language-network reorganization in people with aphasia, although scanner constraints limit overt speech, interactive communication, and assessment under more naturalistic conditions (Griffis et al., 2017; Maloney et al., 2023). Complementary neurophysiological approaches, particularly EEG and functional near-infrared spectroscopy (fNIRS), offer opportunities to examine language processing beyond conventional MRI environments. EEG-based neural tracking of natural speech provides millisecond-level temporal information regarding how the brain follows continuous linguistic input and has been proposed as an ecologically valid biomarker for detecting aphasia (De Clercq et al., 2025). In contrast, fNIRS measures cortical hemodynamic responses with relatively greater feasibility for repeated assessment during overt language tasks, bedside evaluation, and rehabilitation settings because of its portability and tolerance for less constrained communication paradigms (Gilmore et al., 2021; Meier et al., 2023; Lu et al., 2025). Together, these complementary modalities provide information across different temporal and spatial scales and may facilitate the development of clinically applicable brain-network biomarkers that can be evaluated during listening, reading, interactive communication, and semi-naturalistic speech production tasks.

A central methodological challenge is to convert naturalistic language samples into reproducible measures that can be tested clinically and related to neural measures. Although existing studies have linked discourse features to lesion characteristics, white matter integrity, functional activation, and connectivity, they rarely apply a common analytic sequence spanning feature extraction, multivariate profile construction, biomarker comparison, and outcome validation. An ecological biomarker framework is therefore most useful when these links are prespecified and evaluated against meaningful communication outcomes.

The present review therefore focuses specifically on how naturalistic language behaviors can be operationalized as clinically meaningful neurobehavioral phenotypes in post-stroke aphasia. Rather than providing a comprehensive review of all language assessment methods, it examines how ecologically valid communication behaviors, extracted behavioral features, and brain-network biomarkers can be integrated to characterize individual recovery patterns and inform precision rehabilitation. Accordingly, the review has two aims. First, it organizes evidence on naturalistic language measures and their neural correlates within an interacting, multivariate account of post-stroke language function. Second, it proposes a testable workflow in which a defined task yields multilevel behavioral features, these features form a graded patient profile, neural measures are evaluated for convergent, dissociable, or incremental information, and both are tested against prespecified outcomes such as discourse change, communication participation, treatment response, or longitudinal recovery. The framework is intended to complement standardized aphasia assessment.

## 2 Methods: structured search and thematic evidence mapping

### 2.1 Search strategy

This integrative review used a structured literature search and descriptive thematic mapping to organize evidence on naturalistic language assessment, multivariate neuropsychological profiling, and neuroimaging biomarkers in post-stroke aphasia. Scoping-review and evidence-mapping guidance was used to improve search transparency and to structure a heterogeneous literature; the review was not designed to estimate a pooled intervention effect or formally grade intervention efficacy (Tricco et al., 2018; Peters et al., 2020; Campbell et al., 2023).

A descriptive thematic mapping approach was used to organize the heterogeneous literature across task, behavioral-profile, modality, and translational domains. The map was intended to characterize where evidence was concentrated or sparse and to support narrative synthesis, rather than to estimate intervention effects or assign formal certainty ratings (White et al., 2020; Campbell et al., 2023).

A literature search was conducted in PubMed/MEDLINE and the Web of Science Core Collection from database inception to June 2026. Search terms were organized around four core concepts: post-stroke aphasia, naturalistic language assessment, neuropsychological phenotyping or ecological validity, and neuroimaging biomarkers. The search strategy combined controlled vocabulary terms and free-text keywords related to aphasia, stroke, connected speech, discourse, narrative, conversation, spontaneous speech, ecological assessment, neuropsychological assessment, fNIRS, fMRI, EEG, ERP, lesion-symptom mapping, functional connectivity, recovery, and rehabilitation.

The PubMed search strategy was as follows: (“post-stroke aphasia” OR “aphasia after stroke” OR “stroke aphasia” OR (stroke AND aphasia)) AND (“naturalistic language” OR “connected speech” OR discourse OR narrative OR conversation OR “spontaneous speech” OR “language production” OR “language comprehension” OR “functional communication”) AND (assessment OR “ecological validity” OR “neuropsychological assessment” OR phenotype OR phenotyping OR biomarker OR “language recovery”) AND (fNIRS OR “functional near-infrared spectroscopy” OR fMRI OR “functional magnetic resonance imaging” OR EEG OR ERP OR neuroimaging OR “functional connectivity” OR “lesion-symptom mapping” OR “brain network”).

The Web of Science search used the same conceptual blocks adapted to the Topic field. Reference lists of included articles and relevant reviews were manually screened to identify additional studies. Searches were limited primarily to peer-reviewed articles published in English. Key theoretical papers and high-quality reviews were also included when they provided important conceptual, methodological, or translational insights relevant to naturalistic language assessment and neuroimaging biomarkers in post-stroke aphasia.

A supplementary targeted search was conducted through 27 August 2026 using additional terms for cortical tracking, continuous speech, phonological encoding, grammatical processing, syntactic complexity, linguistic prediction/surprisal, and multivariate language features. This search expanded coverage of emerging continuous-speech and real-time language-processing studies, including cortical speech tracking in post-stroke aphasia (Quique et al., 2023; Kries et al., 2024) and recent work on syntactic tracking in aphasia (Rafferty et al., 2026). These studies were incorporated into the narrative synthesis.

### 2.2 Eligibility criteria

Studies were included when they involved individuals with post-stroke aphasia, or findings directly relevant to language recovery after stroke, and addressed one or more of the following content domains:

1 The study population included individuals with post-stroke aphasia, or the findings were directly relevant to language recovery, neuropsychological mechanisms, or brain-network reorganization after stroke.
2 The study investigated naturalistic or ecologically valid language behaviors, including connected speech, discourse production, narrative production or comprehension, conversation, interactive question–answer tasks, spontaneous speech, contextual reading, or functional communication.
3 The study examined language-related behavioral dimensions or neuropsychological profiles, including lexical-semantic access, phonological encoding, grammatical or structural processing, discourse organization, executive-predictive control, speech-motor output, or communication participation.
4 The study reported neuroimaging, neurophysiological, lesion-based, or brain-network evidence relevant to language processing or recovery, including fNIRS, fMRI, EEG/ERP, structural imaging, lesion-symptom mapping, resting-state connectivity, or task-related activation.
5 The article was an original research study, systematic review, scoping review, meta-analysis, methodological paper, or influential theoretical review relevant to the scope of this article.

Studies were excluded if they met any of the following criteria:

1 The study focused exclusively on non-stroke aphasia, such as primary progressive aphasia, traumatic brain injury, brain tumor, or neurodegenerative disease, without data or discussion relevant to post-stroke aphasia.
2 The study examined only isolated single-word naming, repetition, reading aloud, or other decontextualized tasks without addressing ecological validity, naturalistic language behavior, neuropsychological phenotyping, or neural mechanisms.
3 The study focused solely on treatment efficacy without reporting language assessment methods, behavioral phenotypes, neuroimaging findings, or mechanistic implications.
4 The article was a case report, conference abstract, editorial without substantive review content, non-peer-reviewed article, or study for which the full text or sufficient bibliographic information was unavailable.
5 The study was a purely technical neuroimaging or signal-processing report without relevance to aphasia, language function, or clinical neuropsychology.

### 2.3 Data charting and evidence mapping

For each included study, the following information was charted: author, year of publication, study design, sample characteristics, stroke stage, aphasia type or severity, language task, assessment method, neuropsychological construct, neuroimaging or neurophysiological modality, brain regions or networks examined, main findings, clinical implications, and evidence gaps.

The mapped literature was coded into seven pragmatic evidence domains corresponding to Table 1:

1 Conventional aphasia assessment and structured impairment measures;
2 Connected speech and discourse production;
3 Narrative and contextual comprehension;
4 Conversation and functional communication;
5 Brain-network biomarkers, including lesion, structural, task-fMRI, and connectivity measures;
6 Portable neurophysiological/hemodynamic assessment, including EEG and fNIRS;
7 Precision-rehabilitation translation, including prognosis, treatment stratification, target selection, and longitudinal monitoring.

**Table 1:** Thematic evidence map of naturalistic language assessment and brain-network biomarkers in post-stroke aphasia.

Evidence domain	Representative tasks/measures	Profile/network focus	Clinical relevance	Key evidence gap
Conventional aphasia assessment (Sheppard and Sebastian, 2021; Halai et al., 2022; Kim and Yang, 2022; Castro et al., 2023)	WAB, BDAE, naming, repetition, comprehension subtests	Impairment severity and aphasia classification	Diagnosis, severity grading, and treatment planning	Limited ecological validity for everyday communication
Connected speech and discourse (Wilmskoetter et al., 2019; Alyahya et al., 2020; Stark et al., 2021a; Stark et al., 2021b; Ding et al., 2023; Cordella et al., 2024)	Picture description, storytelling, free speech, discourse samples	Lexical-semantic, grammatical, fluency, informativeness, and discourse-organization features	Captures communication-relevant language beyond isolated test items	Heterogeneous elicitation and scoring; limited standardization
Narrative and contextual comprehension (Duncan and Small, 2016; Gleichgerrcht et al., 2016; Quique et al., 2023; Kries et al., 2024)	Story listening, narrative comprehension, paragraph-level processing	Semantic integration, contextual inference, auditory comprehension, and working memory	Assesses extended comprehension and contextual integration	Few naturalistic comprehension studies linked to bedside imaging
Conversation and functional communication (Borovsky et al., 2007; Schumacher et al., 2020; Barberis et al., 2026)	Interactive question-answer tasks, conversational speech, pragmatic communication	Executive-linguistic control, turn-taking, response coherence, and participation	Direct relevance to social participation and daily communication	Underrepresented in post-stroke aphasia neuroimaging
Brain-network biomarkers (Ivanova et al., 2016; Xing et al., 2016; Geranmayeh et al., 2017; Griffis et al., 2017; Sihvonen et al., 2023)	Task activation, resting-state connectivity, structural connectivity, lesion mapping	Residual left language networks, right-hemisphere compensation, bilateral reorganization, and control networks	Recovery prediction, rehabilitation stratification, and target selection	Need longitudinal validation and multimodal integration
Portable EEG/fNIRS assessment (Gilmore et al., 2021; Meier et al., 2023; Quique et al., 2023; Kries et al., 2024; De Clercq et al., 2025)	EEG speech tracking; task/rest fNIRS	Time-resolved linguistic tracking; cortical hemodynamics and connectivity	Repeated or bedside-compatible measurement	Artifact sensitivity, lesion-aware localization, limited standardization and external validation
Precision rehabilitation translation (Hara et al., 2017; Chang et al., 2022; Falconer et al., 2024)	Biomarker-informed therapy, neuromodulation, and longitudinal monitoring	Profile-informed treatment hypotheses and network-guided targets	Individualized rehabilitation planning and outcome monitoring	Few prospective tests of biomarker-guided decisions

For each retained record, coding was performed at the level required to place the study within one or more of these seven domains. The map was descriptive rather than mutually exclusive: a study could contribute to more than one domain when, for example, connected-speech measures were related to lesion location or connectivity. The categories were finalized by comparing the extracted task, behavioral construct, modality, and clinical application fields. Table 1 reports representative studies directly within each domain.

### 2.4 Synthesis approach

Because studies in this field varied substantially in patient characteristics, aphasia stage, language task design, outcome measures, imaging modalities, and analytic methods, a quantitative meta-analysis was not performed. Instead, findings were synthesized narratively and integratively according to evidence domain.

The identification and screening counts were kept distinct from the set used for detailed full-text synthesis. The searches yielded 240 records; 47 duplicates were removed, leaving 193 records for title/abstract screening. Eighty-three records were retained for broad thematic mapping. Full-text PDFs were retrieved and reviewed for 32 prioritized records in two batches; 27 were retained as core or supporting full-text evidence and five were excluded from the core synthesis because they were peripheral to the principal task-behavior-neural links or did not provide completed evidence. Fourteen additional potentially relevant reports could not be retrieved in full and were not used to support detailed mechanistic claims. Figure 1 shows these overlapping levels of evidence, and Supplementary Tables S1 identifies the 27 retained full-text records and their role in the synthesis.

**Figure 1:**
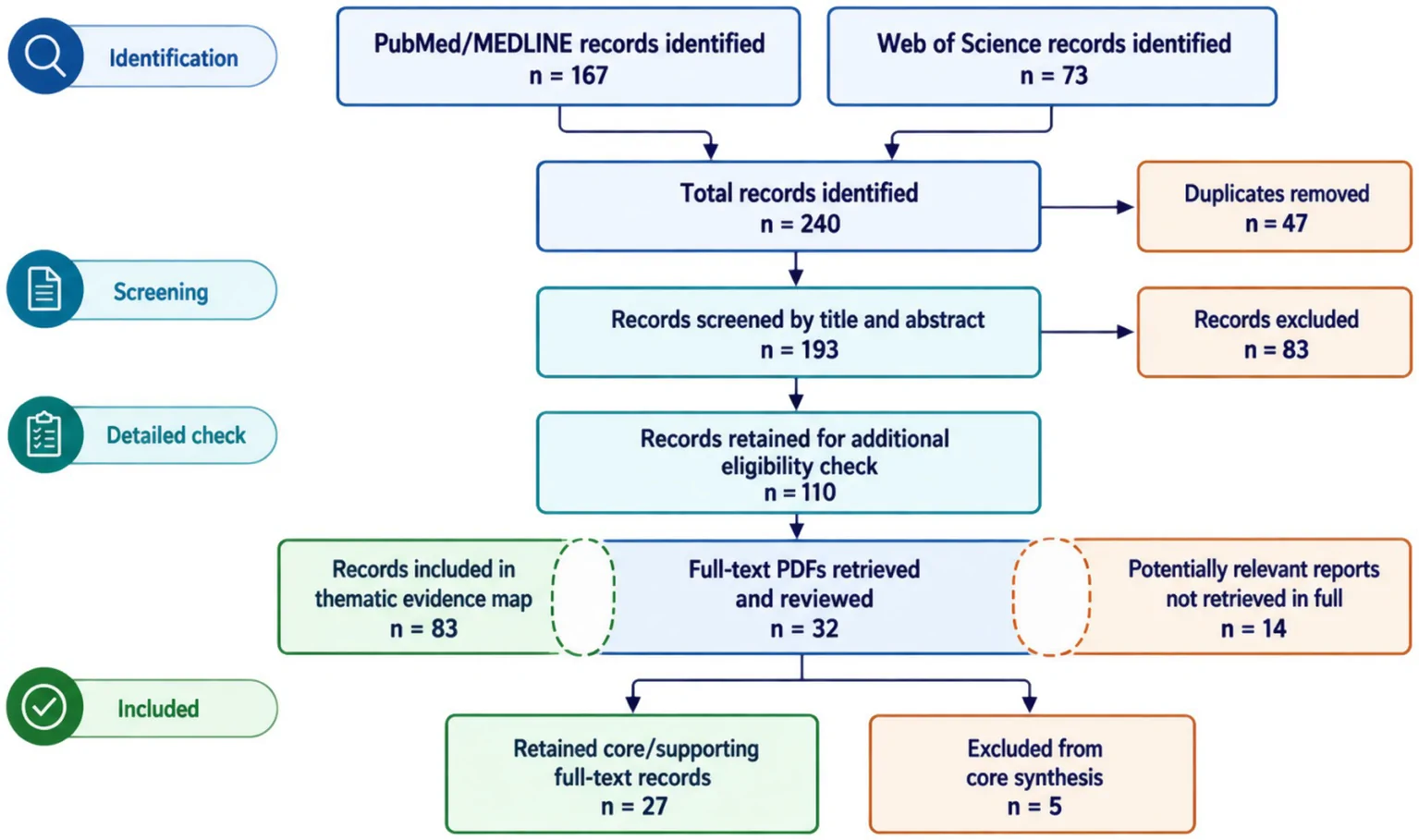
Literature identification, thematic mapping, and targeted full-text synthesis. After duplicate removal, 193 records underwent title/abstract screening and 83 were retained for broad thematic mapping. Full-text retrieval was undertaken for a prioritized, overlapping subset of retained records: 32 PDFs were reviewed, 27 were retained as core or supporting full-text evidence, and five were excluded from the core synthesis; 14 additional prioritized reports could not be retrieved in full. The thematic-map and full-text subsets are therefore not mutually exclusive branches.

Methodological guidance, diagnostic reviews, theoretical neurolinguistic papers, and selected studies outside the 27 retained full-text records were used as supporting literature when needed to define constructs, interpret mechanisms, or discuss methodological limitations. These supporting references were not counted as retained full-text evidence-map records.

The synthesis focused on three questions:

1 Which naturalistic language behaviors have been used to characterize post-stroke aphasia beyond conventional impairment-based assessment?
2 How do these behaviors map onto interacting behavioral profile dimensions and brain-network reorganization after stroke?
3 Which neuroimaging biomarkers are most promising for ecological assessment, recovery prediction, and precision rehabilitation?

The framework was derived as an operational synthesis of the mapped evidence and is implemented through seven steps: (1) define a real-world communication target; (2) select a task that samples that target; (3) extract a reproducible multilevel feature set; (4) construct a graded, multivariate patient profile; (5) test whether neural measures add convergent, dissociable, or incremental information beyond behavioral severity and lesion burden; (6) translate only validated combinations into prognosis or treatment hypotheses; and (7) repeat behavioral and neural measures longitudinally to test change and transfer. Figure 2 illustrates this workflow.

**Figure 2:**
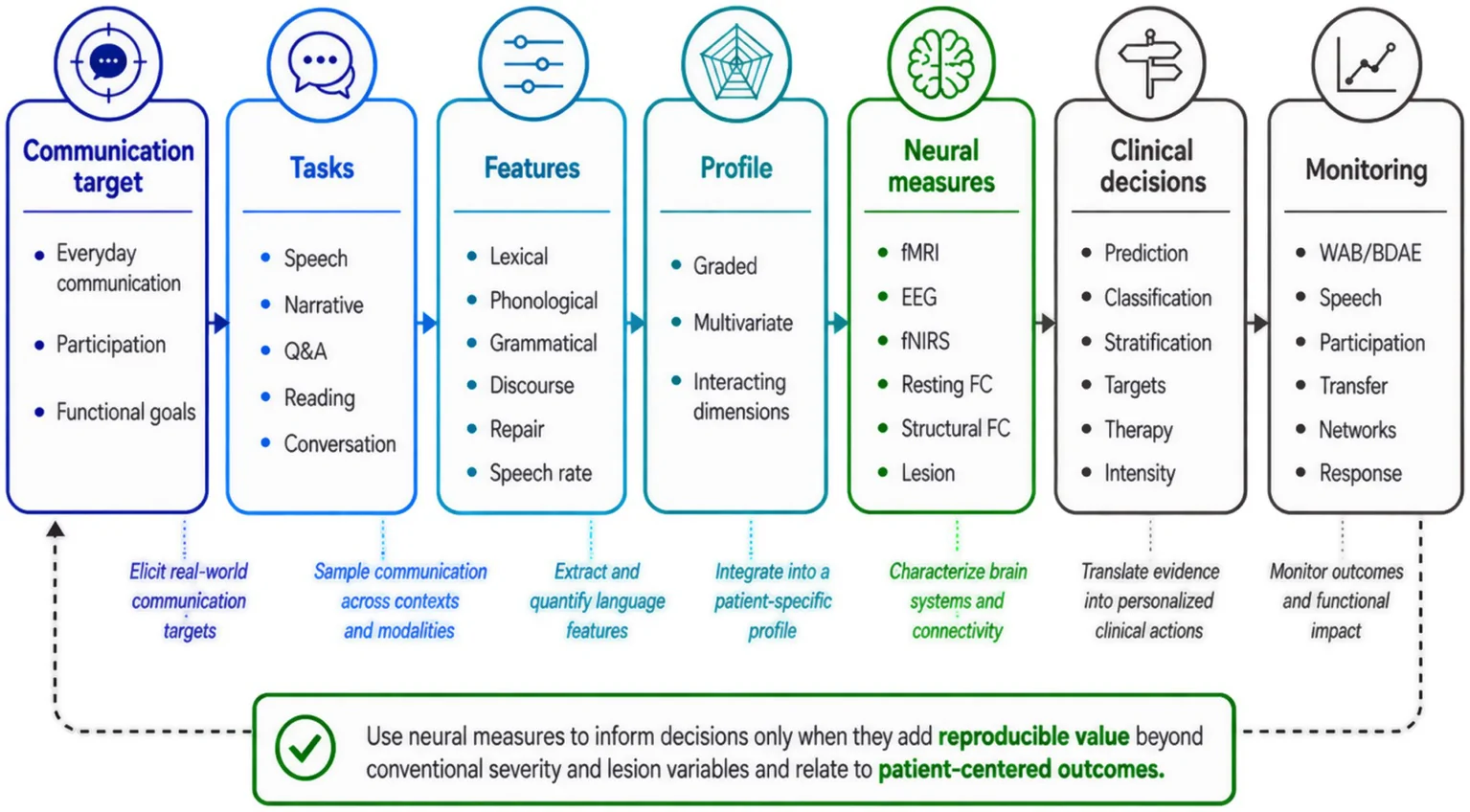
Seven-step operational workflow of the ecological neuropsychological biomarker framework for precision aphasia rehabilitation. The workflow separates the five conceptual layers into seven operational steps: communication target, task selection, feature extraction, multivariate profile construction, neural measurement, clinical decision-making, and longitudinal monitoring. Neural measures advance to clinical use only when they add reproducible value beyond conventional severity and lesion variables and predict patient-centered communication outcomes.

## 3 Evidence map: what has been studied and what remains missing

The evidence map suggests a growing interest in moving beyond impairment-based aphasia assessment toward more ecologically oriented approaches that capture discourse-level and communication-relevant language behaviors. Conventional aphasia batteries, including standardized measures of naming, repetition, comprehension, and spontaneous speech, remain central to diagnosis, severity classification, and treatment planning (Sheppard and Sebastian, 2021; Kim and Yang, 2022). However, these measures provide only a partial representation of the dynamic language demands encountered in real-world communication, particularly when the clinical goal is to understand discourse participation, functional communication, and rehabilitation-relevant change. The major evidence domains identified through this mapping process are summarized in Table 1.

The thematic map summarizes evidence distribution rather than evidence strength. The 83 mapped records characterize breadth across seven domains, whereas the 27 retained full-text records provide the main detailed evidence for Sections 4–6. Because studies could contribute to more than one domain, counts are non-additive; Table 1 and Supplementary Tables S1 provide thematic and record-level links, respectively.

A growing body of evidence has examined connected speech, discourse production, narrative language, and spontaneous speech as naturalistic behavioral markers of aphasia (Alyahya et al., 2020; Stark et al., 2021a; Stark et al., 2021b; Azios et al., 2022; Cordella et al., 2024). These measures capture clinically relevant dimensions including informativeness, lexical diversity, syntactic organization, fluency, coherence, and communicative efficiency, and several studies have linked these features to lesion location, white matter integrity, structural connectivity, and functional activation.

Despite this progress, the evidence remains unevenly distributed. Connected speech and discourse production have received increasing attention, whereas interactive communication, conversational turn-taking, contextual reading, and naturalistic comprehension remain comparatively underrepresented (Stark et al., 2021a; Stark et al., 2021b). Similarly, although fMRI, lesion-symptom mapping, and diffusion-based structural connectivity studies have contributed important insights into the neural substrates of discourse and language recovery (Wilmskoetter et al., 2019; Alyahya et al., 2020; Schumacher et al., 2020; Ding et al., 2023), fewer studies have applied portable and repeatable neuroimaging approaches such as fNIRS during ecologically valid language tasks in post-stroke aphasia (Gilmore et al., 2021; Meier et al., 2023; Lu et al., 2025). This gap is clinically important because bedside-compatible neuroimaging may be particularly suitable for monitoring language-related cortical activation and network reorganization during rehabilitation.

The evidence map therefore indicates three major gaps. First, naturalistic language tasks are not yet standardized across studies, making it difficult to compare findings or establish normative benchmarks (Stark et al., 2021a; Stark et al., 2021b). Second, behavioral measures of discourse and functional communication are rarely integrated with brain-network biomarkers in a unified framework, despite evidence linking discourse features to lesion and connectivity patterns (Wilmskoetter et al., 2019; Alyahya et al., 2020; Schumacher et al., 2020; Ding et al., 2023). Third, few studies have translated naturalistic language biomarkers into clinical decision-making, such as prognosis prediction, rehabilitation stratification, neuromodulation target selection, or longitudinal outcome monitoring (Saur et al., 2006; Falconer et al., 2024). These gaps support the need for an ecological neuropsychological biomarker framework that links naturalistic language behavior, brain-network reorganization, and precision aphasia rehabilitation.

## 4 Naturalistic language behaviors as neuropsychological phenotypes

Structured aphasia tasks remain essential for identifying specific impairments. Naturalistic sampling serves a different purpose: it captures how several linguistic and cognitive demands interact within extended communication. The contrast here is therefore between more and less constrained sampling, not between impairment and real-world processes.

In this review, naturalistic language behavior is treated as a source of graded neuropsychological phenotype information. Rather than assigning a discrete syndrome, a sample is represented by an observable multivariate profile that reflects interactions among cognitive-linguistic operations and the brain networks that support them. Connected speech, narrative production, discourse informativeness, conversational participation, and contextual comprehension can therefore be analyzed as multidimensional expressions of post-stroke language-network disruption. This interpretation is supported by evidence linking discourse-level variables to lesion location, white matter pathways, structural connectivity, and functional activation (Borovsky et al., 2007; Gleichgerrcht et al., 2016; Wilmskoetter et al., 2019; Alyahya et al., 2020; Stark et al., 2021b; Ding et al., 2023; Desjardins et al., 2026; Jacobs et al., 2026).

Operationally, we use phenotype to mean a graded pattern of measurable features rather than a discrete syndrome. The dimensions discussed below are intentionally overlapping. They are descriptive axes for organizing multivariate naturalistic data, not claims that lexical, semantic, syntactic, executive, or motor processes are neurally or computationally independent.

Connected speech is used here as an umbrella term for multi-utterance spoken output under relatively open constraints. Narrative production organizes events around a temporal, causal, or story structure; descriptive speech is more stimulus-bound; responsive speech follows prompts or questions; and conversation is bidirectional and requires turn-taking and interlocutor adaptation. Functional communication refers to successful exchange of personally or socially relevant information, including compensatory non-verbal strategies.

### 4.1 Connected speech and discourse production

Connected speech is useful because it samples lexical access, grammatical encoding, discourse organization, monitoring, and speech-motor output within the same extended communicative goal. Constrained tasks can recruit the same operations, but usually with a narrower response space and less interaction among them.

Recent work has increasingly supported the value of discourse analysis as a multidimensional approach to post-stroke aphasia. An international survey of spoken discourse assessment practices showed that discourse analysis is widely recognized as clinically meaningful, but also highlighted substantial heterogeneity in elicitation methods, scoring procedures, and outcome variables (Stark et al., 2021b). This variability has limited the comparability of findings across studies and underscores the need to define discourse features according to interpretable neuropsychological constructs.

Several studies have begun to address this gap by linking discourse-level performance to neural correlates. Proposed a unified model of post-stroke language deficits that incorporated discourse production and demonstrated that connected speech variables could be integrated with more traditional language measures to reveal meaningful dimensions of aphasic impairment and their neural correlates (Alyahya et al., 2020). More recent studies have further decomposed discourse into macrostructural and microstructural features, including coherence, informativeness, lexical diversity, syntactic complexity, and fluency, and have related these features to white matter integrity and lesion distribution (Alyahya et al., 2026; Desjardins et al., 2026; Jacobs et al., 2026). These findings suggest that discourse impairment should not be treated as a single deficit but rather as a set of dissociable phenotypes reflecting different language and control processes.

This view is also consistent with existing behavioral discourse frameworks. Dipper et al. (2021), for example, emphasized that discourse assessment should integrate microstructural and macrostructural features together with functional communication demands. Building on this perspective, the present review extends discourse assessment toward a neurobehavioral framework in which discourse-related features are linked to distributed brain-network biomarkers and prospective rehabilitation decisions.

A particularly important implication is that connected speech may provide a bridge between impairment-level language scores and real-world communication outcomes. For example, discourse-based measures can capture lexical retrieval efficiency, content word production, narrative organization, and communicative adequacy in ways that are more ecologically valid than isolated naming tasks. Studies of discourse content have shown that impaired informativeness is associated with frontal white matter damage, supporting the idea that discourse production depends not only on classical perisylvian language regions but also on frontal systems involved in selection, organization, and control (Ding et al., 2023). Similarly, lesion-based studies of conversational speech production have demonstrated that natural speech deficits are associated with distributed lesion patterns rather than a single cortical language region (Borovsky et al., 2007). These findings support the use of connected speech as a behavioral phenotype of distributed language-network disruption.

### 4.2 Narrative production and semantic organization

Narrative production is a specific form of connected speech that requires the speaker to construct meaning across events, maintain temporal and causal coherence, select relevant content, and organize information into a communicatively interpretable sequence. It therefore provides a useful paradigm for evaluating semantic integration, discourse planning, lexical-semantic access, and executive-linguistic control.

Evidence from narrative speech studies indicates that different semantic categories and discourse features may depend on partially separable neural systems. Gleichgerrcht et al. showed that action and object representations during narrative speech in post-stroke aphasia were supported by distinct neural systems, suggesting that narrative language can reveal category-specific semantic organization during naturalistic production (Gleichgerrcht et al., 2016). Further linked several complementary discourse measures to specific neuroanatomical structures: lexical diversity captures the range or distribution of words, lexical sophistication reflects the use of less frequent or more specific words, informativeness indexes task-relevant content, and coherence describes local or global organization around the intended topic (Wilmskoetter et al., 2019). Although their exact operationalization varies across studies, these measures show how narrative analysis can move beyond global severity to characterize more specific lexical-semantic and discourse-level difficulties.

Beyond these feature-level associations, narrative production is also related to large-scale network organization. Duncan and Small reported that increased modularity of resting-state networks was associated with improved narrative production during aphasia recovery (Duncan and Small, 2016). Narrative performance may therefore provide a functional readout of how residual language regions, control networks, and compensatory systems operate together after stroke, although this association does not diminish the contribution of focal lesion characteristics.

### 4.3 Descriptive, responsive, and conversational speech

Naturalistic production encompasses descriptive, responsive, and conversational speech, which differ in stimulus constraint and in the degree of listener adaptation, turn-taking, and repair they require. Recent behavioral and neural evidence indicates that descriptive and responsive speech are related but non-identical constructs, with different demands on lexical retrieval, discourse organization, cognitive control, and neural resources (Barberis et al., 2026). Accordingly, performance in structured picture description may not generalize to responsive or interactive communication, where contextual and pragmatic cues can either increase communicative demands or provide compensatory support.

Functional communication is additionally shaped by verbal, non-verbal, and structural factors (Schumacher et al., 2020). Conversational and functional measures therefore capture the combined influence of language ability, cognitive control, communicative strategies, and environmental support, providing higher-order phenotypic information that complements impairment-level scores.

### 4.4 Interacting linguistic and cognitive phenotype dimensions

A strictly modular partition of lexical, semantic, and syntactic phenotypes would be difficult to justify from contemporary neurolinguistics. Naturalistic language engages tightly coupled representations, and recent work shows distributed sensitivity to syntax and semantics across the language network rather than a clean anatomical separation (Shain et al., 2024). Work on cortical tracking likewise shows that linguistic structure is shaped by lexical-semantic content and statistical predictability (Coopmans et al., 2022; Slaats et al., 2024). Accordingly, the dimensions below should be interpreted as partially separable measurement axes within a multivariate profile, not as independent modules.

Naturalistic language samples can be summarized along several interacting dimensions. One dimension is lexical-semantic access, which may be characterized by reduced word output, word-finding pauses, circumlocution, low content-word fluency, and limited lexical diversity. Connected speech is useful because lexical access is repeatedly sampled under ongoing communicative demands. Discourse lexical features such as diversity and sophistication can index residual lexical-semantic capacity (Wilmskoetter et al., 2019).

A discourse-semantic dimension is reflected in reduced informativeness, impaired thematic coherence, weak content organization, and difficulty integrating contextual meaning across utterances. Narrative and discourse tasks are particularly informative because they require selection and organization of relevant semantic information across multiple propositions. Studies of narrative speech and discourse content suggest that these difficulties can relate to left posterior language regions as well as frontal white matter systems involved in controlled retrieval and selection (Gleichgerrcht et al., 2016; Ding et al., 2023).

A grammatical/structural dimension can be expressed as reduced sentence complexity, grammatical omissions, simplified utterance structure, or disrupted morphosyntactic planning. Lesion-symptom work indicates that components of grammatical production rely on distributed frontotemporal and dorsal-stream systems (Gleichgerrcht et al., 2021). These findings support decomposing grammatical performance into measurable operations and error patterns rather than treating syntax as a unitary phenotype.

An executive-predictive control dimension captures selection among competing alternatives, topic maintenance, inhibition of irrelevant information, error monitoring, contextual adaptation, and the use of expectations during comprehension or production. These processes become especially relevant when conventional language scores do not fully explain communication difficulty. Domain-general medial prefrontal systems have been implicated in post-stroke language recovery (Geranmayeh et al., 2017). Less constrained speech tasks may therefore expose variation in the interaction between language processing and cognitive control.

A speech-motor output dimension captures articulatory planning, motor-speech execution, fluency regulation, and the transformation of phonological plans into overt speech. Casilio et al. identified paraphasia, logopenia, agrammatism, and motor-speech dimensions in naturalistic language production after stroke and related them to partially distinct but overlapping neuroanatomical correlates (Casilio et al., 2025). This multidimensional account is useful for separating speech-language features while retaining their interaction within an individual profile.

Phonological encoding is treated as a cross-cutting dimension rather than being absorbed into motor speech. It includes selection and sequencing of phonological word forms, phonemic paraphasias, phonotactic errors, and auditory-phonological representations. These features can affect lexical retrieval, repetition, connected speech, and speech-motor output, and should therefore be quantified separately when the task permits.

Grammar is similarly non-monolithic. Lesion-symptom work has demonstrated a double dissociation between agrammatism, characterized by simplification and omission and associated more strongly with frontal damage, and paragrammatism, characterized by morphosyntactic selection or structural errors and associated more strongly with posterior temporal damage (Matchin et al., 2020; Matchin et al., 2023). Recent EEG work further shows that the timing of neural alignment with distinct syntactic computations relates differently to online comprehension in aphasia (Rafferty et al., 2026). A single ‘syntactic phenotype’ should therefore be decomposed into measurable error types or computations whenever data allow.

Predictive processing is another cross-cutting feature. Word surprisal, contextual expectation, and dependency-related prediction can influence lexical access, structure building, and comprehension; naturalistic EEG work in post-stroke aphasia has begun to show altered tracking of acoustic and word-level linguistic representations (Quique et al., 2023; Kries et al., 2024). We therefore place prediction within the multivariate phenotype architecture rather than treating it as a separate clinical subtype.

### 4.5 Implications for ecological assessment and precision rehabilitation

Naturalistic samples complement standardized batteries: conventional tests quantify severity and selected impairments, whereas less constrained samples show how difficulties combine under communicative load. The same patient can therefore be described by both conventional scores and a graded naturalistic-language profile.

Multivariate profiles may eventually improve clinical stratification, but this requires prospective testing. Patients with similar global severity can show different mixtures of lexical-semantic, phonological, grammatical, discourse, executive-control, and motor-speech difficulty. These mixtures should be modeled continuously, and treatment matching should be tested empirically rather than assumed.

Multivariate naturalistic profiles also provide a behavioral layer for biomarker development. Their features can be related to fMRI, EEG, fNIRS, lesion, structural-connectivity, and resting-state measures to test how communication difficulty relates to network organization.

Finally, these phenotypes may support longitudinal monitoring and precision rehabilitation. Emerging evidence suggests that acute white matter characteristics can predict longitudinal recovery of naming and connected speech (Desjardins et al., 2026), and that network organization is associated with narrative recovery (Duncan and Small, 2016). Such findings indicate that naturalistic language measures could be used not only to describe current impairment but also to predict recovery trajectories, guide treatment selection, and monitor rehabilitation-induced change. In future research, integrating naturalistic language sampling with complementary portable measures such as EEG and fNIRS may help establish ecologically valid biomarkers for individualized aphasia rehabilitation.

## 5 Neuroimaging biomarkers of naturalistic language processing after stroke

Naturalistic language behaviors become most informative when they are linked to interpretable neural mechanisms. Accordingly, this section moves from residual left-hemisphere systems and conditional right-hemisphere support to domain-general control, network connectivity, and portable measures of language processing.

Evidence from fMRI, structural MRI, diffusion imaging, lesion-symptom mapping, EEG/ERP, and fNIRS suggests that language recovery after stroke is best understood as a distributed network process. The surviving left-hemisphere language system remains critical for recovery, but right-hemisphere homologues, bilateral reorganization, frontoparietal control systems, white matter integrity, and functional connectivity also influence recovery trajectories (Cao et al., 1999; Ivanova et al., 2016; Xing et al., 2016; Geranmayeh et al., 2017; Griffis et al., 2017; Schumacher et al., 2020; Wilson and Schneck, 2021; Barbieri et al., 2023; Maloney et al., 2023; Sihvonen et al., 2023). These findings are particularly relevant for naturalistic language paradigms because discourse production, narrative comprehension, and conversation require the coordinated recruitment of semantic, syntactic, executive, auditory, motor, and social-pragmatic processes. Thus, ecological language biomarkers should be conceptualized at the level of networks rather than isolated cortical regions. To illustrate this network-level perspective, Figure 3 summarizes how naturalistic language behaviors can be mapped onto neuropsychological phenotypes, brain-network mechanisms, and candidate biomarkers.

**Figure 3:**
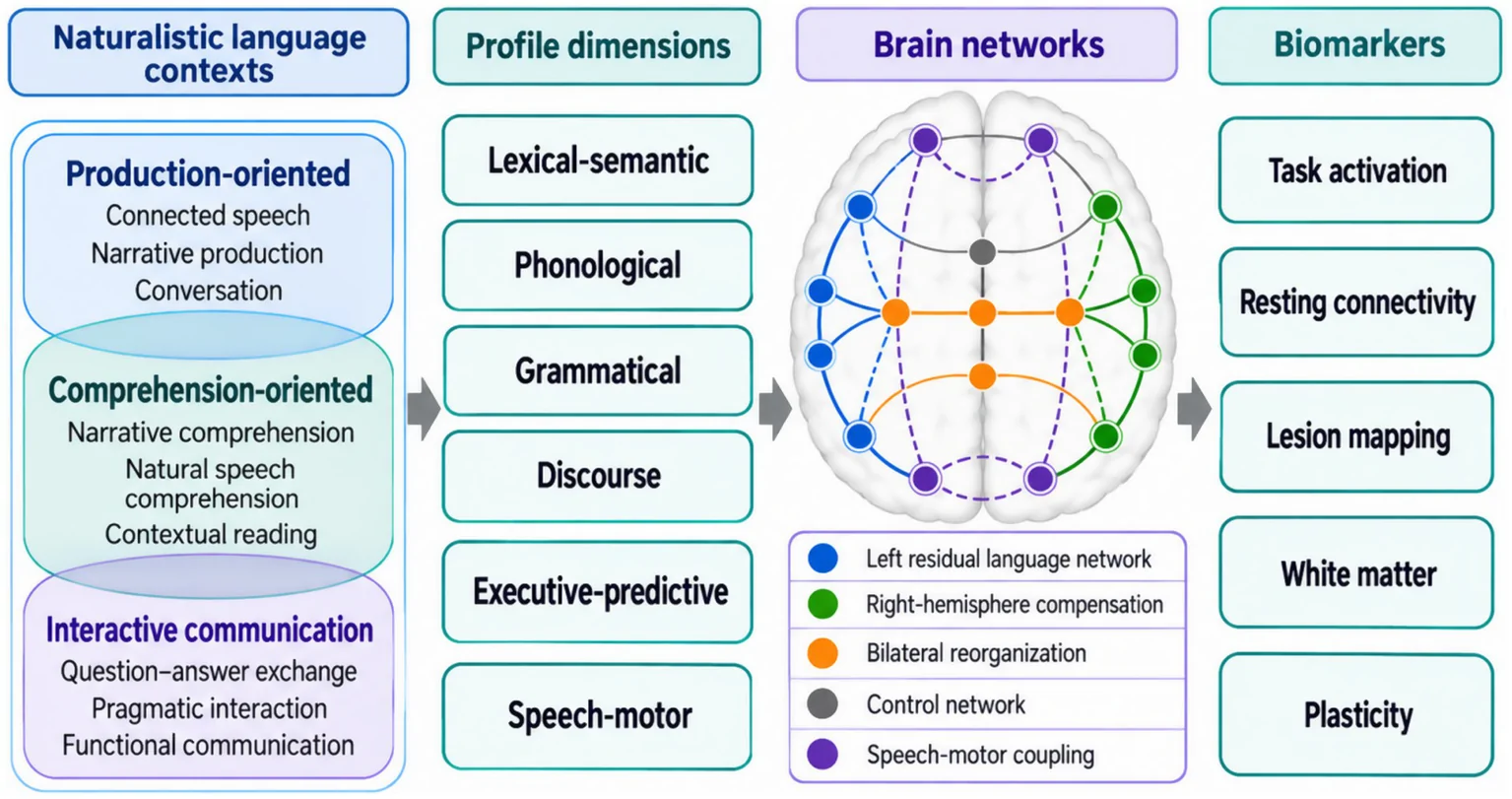
Naturalistic language contexts, profile dimensions, brain networks, and candidate biomarkers in post-stroke aphasia. Naturalistic language contexts are shown as overlapping production-oriented, comprehension-oriented, and interactive communication domains rather than mutually exclusive task classes. These contexts yield multilevel features that can be organized into interacting profile dimensions and related to distributed language, control, compensatory, and speech-motor networks. Candidate biomarkers include task-related activation, resting connectivity, lesion-symptom mapping, white matter integrity, and longitudinal network plasticity. Network nodes and connections are schematic and illustrate network-level relationships rather than precise anatomical localization. The posterior extension of the speech-motor pathway denotes task-dependent visual-perceptual integration in visually cued language paradigms rather than a core occipital speech-motor node. The profile dimensions are shown separately for readability but are modeled as overlapping rather than independent modules; lexical-semantic, phonological, grammatical, discourse, executive-predictive, and speech-motor features may covary within an individual sample.

### 5.1 Left residual language networks

A central question in post-stroke aphasia is whether recovery is primarily supported by restoration of residual left-hemisphere language networks or by compensatory recruitment of alternative systems. The available evidence suggests that preserved or re-engaged left-hemisphere regions remain essential, especially when residual tissue is available within core language networks. A systematic review of neuroplasticity in post-stroke aphasia concluded that there is modest evidence for a return of function in left-hemisphere language regions over time, while evidence for large-scale dynamic reorganization into entirely new systems remains less compelling (Wilson and Schneck, 2021). This finding is important for ecological assessment because it suggests that naturalistic language performance may depend strongly on the integrity and reactivation of residual language networks rather than on complete functional substitution.

This conclusion is supported across complementary methods rather than by a single imaging literature. Lesion-symptom mapping, multivariate behavioral-neural models, diffusion imaging, and longitudinal functional imaging all show that residual left frontotemporal and temporoparietal tissue and its connections constrain language performance and recovery (Saur et al., 2006; Ivanova et al., 2016; Alyahya et al., 2020; Stockert et al., 2020; Halai et al., 2022; Matchin et al., 2022; Turken and Dronkers, 2011). The exact contribution depends on the linguistic measure and lesion distribution, which is why naturalistic outcomes should be analyzed alongside rather than instead of constrained tasks.

The left residual language network includes inferior frontal regions, posterior superior and middle temporal regions, temporoparietal areas, angular and supramarginal gyri, and dorsal and ventral white matter pathways. These regions support distinct but interacting operations, including lexical retrieval, semantic integration, phonological processing, syntactic organization, auditory comprehension, and speech production. In chronic post-stroke aphasia, activation of canonical semantic networks has been shown to support residual language function, including semantic comprehension, naming, and fluency (Griffis et al., 2017). This supports the view that preserved portions of the normal language network continue to provide the neural substrate for recovery.

Importantly, naturalistic language tasks may be especially sensitive to residual network capacity. Single-word naming or repetition can sometimes be supported by narrow task-specific strategies, whereas connected speech requires sustained access to lexical-semantic knowledge, sentence planning, discourse organization, and output monitoring. Functional MRI studies using overt or free discourse paradigms have shown that naturalistic speech production can be studied with task designs that are feasible for people with aphasia, thereby extending neuroimaging beyond highly constrained word- or sentence-level tasks (Maloney et al., 2023). Such paradigms are particularly valuable because they allow imaging results to be linked to discourse-level variables that are closer to functional communication.

Lesion-symptom mapping further shows that language deficits are associated not only with cortical damage but also with disruption of distributed networks and white matter pathways. Diffusion imaging studies have demonstrated that major language-related tracts, including the arcuate fasciculus, superior longitudinal fasciculus, inferior fronto-occipital fasciculus, inferior longitudinal fasciculus, and uncinate fasciculus, contribute to language processing and aphasia profiles (Ivanova et al., 2016; Mochizuki et al., 2023). For naturalistic language processing, these pathways may be especially relevant because discourse and narrative production require long-range integration between frontal control systems, temporal semantic regions, and articulatory-motor systems.

In Figure 3, the posterior extension of the speech-motor coupling network is intended to represent task-dependent integration with posterior visual-perceptual regions during visually cued language tasks, such as picture description or reading, rather than a core occipital component of the speech-motor system.

Residual left-hemisphere activation should be interpreted together with lesion extent, white matter integrity, and task demand. Similar behavioral scores can arise from different combinations of preserved temporal, frontal, and dorsal-stream systems, making multimodal and task-sensitive interpretation preferable to a single activation marker.

### 5.2 Right-hemisphere compensation and bilateral reorganization

The role of the right hemisphere in post-stroke aphasia remains one of the most debated issues in the field. Early functional imaging studies reported increased right-hemisphere activation during language tasks in recovered aphasic patients and interpreted this as evidence of compensatory recruitment (Cao et al., 1999). Subsequent work complicated this view by showing that right-hemisphere engagement may be adaptive, inefficient, or maladaptive depending on lesion size, residual left-hemisphere function, time post-stroke, task demand, and aphasia severity.

Studies of right-hemisphere structure provide important support for a nuanced interpretation. Right-hemisphere grey matter structure has been associated with language outcomes in chronic left-hemisphere stroke, indicating that right-hemisphere tissue characteristics may influence recovery (Xing et al., 2016). More recent structural connectivity work has shown that right-hemisphere white matter connectivity can predict post-stroke language recovery, suggesting that right-hemisphere networks may support language function when left-hemisphere pathways are severely compromised (Sihvonen et al., 2023). These findings are relevant to naturalistic language assessment because discourse and conversation may place higher demands on compensatory resources than isolated language tasks.

The direction of these associations is not uniform. Xing et al. reported that greater grey-matter volume in right temporoparietal regions was positively associated with spontaneous speech, naming, and repetition after controlling for left-hemisphere lesion burden (Xing et al., 2016). By contrast, Sihvonen et al. found that stronger right-hemisphere structural connectivity was negatively associated with language production but positively associated with comprehension, while interhemispheric connectivity was strongly related to comprehension (Sihvonen et al., 2023). These findings argue against treating ‘more right-hemisphere recruitment’ as a unitary marker of better recovery.

At the same time, right-hemisphere recruitment should not be interpreted as universally beneficial. In chronic post-stroke aphasia, activation within bilateral semantic networks has been associated with language performance, but right frontal activations may have different behavioral implications depending on the degree of left-hemisphere damage (Griffis et al., 2017). This supports a conditional compensation model: right-hemisphere recruitment may be beneficial when left-hemisphere damage is extensive and residual language networks are insufficient, but less efficient or even maladaptive when the left hemisphere retains substantial language capacity.

Bilateral reorganization also appears in treatment-induced plasticity. Large-scale fMRI work has shown that treatment-induced neural reorganization in aphasia can be language-domain specific, with different language treatments leading to different patterns of neural change (Barbieri et al., 2023). This finding is important for precision rehabilitation because it suggests that biomarkers should not simply classify activation as left- or right-lateralized. Instead, they should identify whether a specific pattern of reorganization supports the language domain being targeted, such as naming, sentence processing, spelling, discourse organization, or functional communication.

Right-hemisphere and bilateral biomarkers are most informative when tied to discourse-level outcomes. Naturalistic speech increases demands on attention, control, pragmatic processing, and motor planning; associations with connected speech, coherence, or participation may therefore help distinguish adaptive support from nonspecific overactivation.

### 5.3 Multiple-demand and executive-control networks

Language recovery also depends on domain-general processes, including attention, working memory, selection, monitoring, inhibition, and planning. These functions are supported by frontoparietal and medial frontal systems that may become especially important when damaged language networks operate inefficiently.

Evidence from neuroimaging supports this view. Domain-general subregions of the medial prefrontal cortex have been shown to contribute to recovery of language after stroke (Geranmayeh et al., 2017). This finding is especially relevant for naturalistic language assessment because connected speech and conversation require more than access to linguistic representations. They require speakers to monitor whether their output is coherent, select relevant information, suppress irrelevant responses, and adapt to listener feedback. Therefore, executive-linguistic control may be a key intermediate phenotype linking naturalistic speech behavior to brain network function.

Functional communication studies also suggest that real-world communication relies on verbal, non-verbal, and structural factors, rather than language impairment alone (Schumacher et al., 2020). This provides further support for incorporating executive-control and domain-general networks into biomarker models. A person with aphasia may show a relatively preserved naming score but poor conversational participation because of impaired discourse planning, reduced cognitive flexibility, poor self-monitoring, or difficulty managing communicative breakdowns. Conversely, preserved control networks may help patients compensate for lexical or syntactic deficits during naturalistic interaction.

These language-control interactions also depend on large-scale network organization. Resting-state network modularity has been associated with improved narrative production during aphasia recovery (Duncan and Small, 2016), suggesting that discourse outcomes reflect coordination across distributed systems rather than focal activation alone. Multimodal analyses that combine lesion burden, structural pathways, and functional connectivity may therefore help explain why patients with similar lesion locations show different communication outcomes (Keator et al., 2021; Kristinsson et al., 2025). The critical test is not whether a connectivity metric merely correlates with narrative performance, but whether it explains reproducible variance beyond lesion burden and conventional language scores and predicts longitudinal change.

Taken together, these findings support the inclusion of domain-general control networks in ecological biomarker models. Frontal and frontoparietal measures may help distinguish primary linguistic impairment from communication difficulty driven mainly by monitoring, planning, or control; time-resolved measures of acoustic and linguistic processing are considered in the following section.

### 5.4 Neural tracking and complementary portable biomarkers

Table 2 summarizes representative neuroimaging and neurophysiological modalities and their complementary roles in ecological aphasia assessment, including lesion characteristics, structural connectivity, functional activation, and network-level alterations. Within this framework, fMRI and lesion-based methods provide spatial and anatomical information, EEG captures millisecond-scale electrophysiological dynamics during continuous speech, and fNIRS provides spatially localized measures of superficial cortical hemodynamics during tasks that are difficult to implement in fMRI. This complementarity is particularly useful for naturalistic paradigms because it links when linguistic information is processed with where task-related cortical responses occur.

**Table 2:** Complementary neuroimaging and neurophysiological modalities for ecological aphasia assessment.

Modality	Main readout	Ecological strengths	Key limitations	Best-suited applications
fMRI	Task-related BOLD activation, lateralization, treatment-induced change	High spatial resolution; established mapping of residual and compensatory language networks	Scanner noise and motion constraints; limited feasibility for overt interaction and repeated bedside use	Residual/compensatory network mapping, controlled discourse paradigms, treatment-induced reorganization
fNIRS	Indirect cortical hemodynamics: HbO/HbR activation, lateralization, resting-state connectivity	Portable; relatively simple preparation; no conductive gel; tolerable for repeated or bedside measurement; spatially localized sampling of superficial cortex	Indirect vascular signal; superficial sensitivity; motion/systemic confounds; regional interpretation in lesioned brains benefits from individual anatomical co-registration	Repeated cortical hemodynamic measurement, semi-naturalistic tasks, and task/rest connectivity when fMRI is impractical
EEG/ERP	Electrical neural activity: oscillations, ERPs, temporal response functions, and speech tracking	Direct electrophysiological signal; millisecond temporal resolution; continuous-speech tracking; portable systems available	Limited source localization; facial/jaw and movement artifacts can affect overt speech; lesion anatomy complicates generic source models	Continuous-speech listening, prediction and linguistic-feature tracking, and time-resolved comprehension dynamics
Lesion-symptom mapping	Associations between lesion location/extent and language deficits	Direct structure-behavior mapping for connected speech and discourse phenotypes	Often cross-sectional; dependent on lesion coverage and sample size; cannot capture dynamic compensation	Lesion correlates of connected speech, syntax, semantics, and functional communication
DTI/structural connectivity	White matter integrity, tract disruption, structural connectivity	Characterizes long-range dorsal/ventral language pathways and structural reserve	Tractography is sensitive to preprocessing and can be difficult in lesioned brains; no real-time function	Recovery prediction, tract integrity, and right-hemisphere structural compensation
Resting-state functional connectivity	Intrinsic connectivity, network modularity, interhemispheric coupling	No active task required; captures large-scale network organization and recovery potential	Affected by motion, vigilance, lesion location, and preprocessing; clinical specificity remains limited	Therapy-response prediction, network reorganization, and narrative recovery
Multimodal integration	Behavioral, lesion, structural, functional, and neurophysiological markers	Integrates behavioral profiles with lesion, structural, functional, and neurophysiological information	Requires larger samples, harmonized protocols, advanced modeling, and external validation	Precision stratification, prognosis, therapy selection, and longitudinal outcome monitoring

Natural-speech EEG has moved beyond envelope tracking alone. Studies have reported altered tracking of the speech envelope in post-stroke aphasia (Quique et al., 2023), extended continuous-speech models to acoustic and linguistic representations such as word-level surprisal (Kries et al., 2024), and demonstrated individual-level aphasia detection during story listening (De Clercq et al., 2025). Related ERP and tracking approaches can also characterize the timing of higher-order linguistic computations. A single continuous stimulus may therefore yield complementary indices of acoustic encoding, lexical prediction, and structural processing within auditory and language networks.

fNIRS complements this temporal information by supporting repeated and bedside measurement of cortical activation and connectivity. Pilot studies have examined language and domain-general processing in aphasia (Gilmore et al., 2021), resting-state connectivity during acute and subacute recovery (Meier et al., 2023), and cognitive interference during Stroop-type tasks (Lu et al., 2025). Together, these applications show how portable hemodynamic measures can sample language and control processes across clinically relevant tasks and recovery stages.

Overall, ecological biomarkers should be interpreted at several linked levels: residual left-hemisphere systems support preserved language operations; right-hemisphere and bilateral systems may provide conditional support; control networks contribute to executive-predictive demands; connectivity constrains communication among these systems; and portable EEG/fNIRS characterize complementary temporal and spatial aspects of task processing. Their clinical value depends on whether they improve prognosis or treatment decisions beyond established clinical variables.

## 6 An ecological neuropsychological biomarker framework for precision aphasia rehabilitation

The proposed framework operationalizes the transition from naturalistic language sampling to clinically testable brain-behavior hypotheses. Each application begins with a prespecified communication outcome, a task selected to sample that outcome, and a defined set of behavioral and neural variables.

Naturalistic samples are converted into multilevel features such as retrieval latency, phonological errors, lexical diversity, informativeness, grammatical error patterns, coherence, repair behavior, and speech rate. These features form a graded patient profile that can be tested against lesion and neural measures for convergent, dissociable, or incremental information.

The framework retains five conceptual layers—naturalistic task, neuropsychological profile, brain-network biomarker, clinical decision, and outcome monitoring—but implements them through the seven operational steps shown in Figure 2: communication target, task selection, feature extraction, profile construction, neural measurement, clinical decision-making, and longitudinal monitoring. The links are evaluated explicitly: task-to-profile asks which features covary or dissociate; profile-to-biomarker tests whether neural measures add information beyond lesion burden and conventional severity; biomarker-to-decision tests whether this added information improves a prespecified prediction or treatment hypothesis; and decision-to-monitoring tests whether expected changes occur and transfer to communication participation.

### 6.1 Naturalistic task layer

The first layer comprises tasks that sample extended or interactive communication, including connected speech, narrative, conversation, question-answer exchange, contextual reading, story comprehension, and real-world communication samples. Elicitation may use picture description, story retelling, interviews, comprehension tasks, or clinician-patient interaction.

Task choice changes the distribution and interaction of processing demands rather than switching individual processes on or off. Naming offers stronger experimental control over lexical retrieval, whereas connected speech permits lexical, grammatical, discourse, executive, and motor features to be quantified together. The framework therefore treats constrained and naturalistic tasks as complementary.

Evidence from discourse and naturalistic language studies supports the clinical value of these tasks. Connected speech and discourse measures have been shown to capture language dimensions not fully reflected by single-word naming or conventional aphasia severity scores (Alyahya et al., 2020; Stark et al., 2021b). Discourse informativeness, lexical diversity, syntactic complexity, and functional communication may reveal clinically important residual abilities or limitations that are obscured by global impairment scores (Wilmskoetter et al., 2019; Schumacher et al., 2020; Ding et al., 2023). Therefore, the naturalistic task layer provides the behavioral foundation for ecological phenotyping.

In practice, naturalistic tasks should complement standardized batteries. A brief connected-speech sample, contextual comprehension task, and functional communication measure can add information about how impairment affects communication without replacing diagnostic testing.

### 6.2 Neuropsychological profile layer

The second layer represents each patient as a multivariate profile rather than a categorical subtype. Core dimensions include lexical-semantic access, phonological encoding, grammatical/structural encoding, discourse-semantic organization, executive-predictive control, speech-motor output, and functional communication. These dimensions are intentionally non-orthogonal and may be modeled jointly.

A lexical-semantic dimension may combine content-word retrieval, lexical diversity, and context-sensitive choice; a phonological dimension may index paraphasias and sequencing; grammar should distinguish omission/simplification from selection or structural errors; discourse can index coherence and event structure; executive-predictive control can index repair, interference resolution, and contextual prediction; and speech-motor measures can quantify rate and articulatory breakdown.

The executive-predictive control dimension is particularly relevant to precision rehabilitation. Some patients may retain partial lexical or grammatical capacity but use it inefficiently in conversation because of impaired selection, monitoring, inhibition, planning, or contextual prediction. This dimension is consistent with evidence that domain-general control systems contribute to language recovery after stroke (Geranmayeh et al., 2017). A speech-motor dimension can separately capture reduced fluency, articulatory-planning difficulty, motor-speech involvement, and disrupted phonological-to-motor transformation. Recent multidimensional analyses of naturalistic production provide an empirical basis for distinguishing these features while modeling them jointly (Casilio et al., 2025).

Recent work has also shown that discourse and lesion features can be combined using machine-learning approaches to estimate conventional aphasia severity (Riccardi et al., 2024). Their clinical value must be demonstrated prospectively. A key test is whether multivariate naturalistic features improve prediction of treatment response or communication participation beyond conventional battery scores before they are used for treatment selection.

### 6.3 Brain-network biomarker layer

The third layer links multivariate profiles to candidate biomarkers from fMRI, EEG/ERP, fNIRS, structural MRI, diffusion imaging, lesion-symptom mapping, and resting-state connectivity (Wilson et al., 2019). The aim is to identify which neural measures explain or constrain specific performance dimensions, not to define a single aphasia biomarker.

The left residual language network remains a central biomarker domain. Recovery is often associated with preserved or re-engaged left-hemisphere language regions and pathways, including inferior frontal, posterior temporal, temporoparietal, and dorsal or ventral white matter systems (Ivanova et al., 2016; Griffis et al., 2017; Wilson and Schneck, 2021). These networks may be particularly relevant for lexical, semantic, syntactic, and phonological phenotypes. However, left-hemisphere residual function should be interpreted in relation to lesion location, lesion volume, white matter integrity, and task demands.

Right-hemisphere compensatory networks represent a second biomarker domain. Right-hemisphere activation or structural connectivity may support recovery in some patients, especially when left-hemisphere damage is extensive (Xing et al., 2016; Sihvonen et al., 2023). At the same time, right-hemisphere recruitment is not uniformly beneficial and may reflect inefficient compensation in some contexts. Therefore, the framework treats right-hemisphere involvement as conditional rather than inherently adaptive or maladaptive.

A third domain is the frontoparietal or multiple-demand control network. Naturalistic language tasks require sustained attention, working memory, response selection, monitoring, and cognitive flexibility. These processes are essential for connected speech and conversation, particularly when the core language system is damaged. Medial prefrontal and other domain-general control regions have been implicated in language recovery after stroke (Geranmayeh et al., 2017). This suggests that executive-linguistic control phenotypes may be linked to control-network biomarkers rather than to classical language regions alone.

Motor-speech coupling is another biomarker domain. Speech production requires coordination among phonological encoding, articulatory planning, premotor and supplementary motor systems, and motor execution. Task-related activation and pathway-level connectivity may therefore help distinguish impaired linguistic formulation from difficulty transforming a planned utterance into overt speech.

Connectivity measures describe how residual language, right-hemisphere homologous, control, auditory, and speech-motor systems communicate as a network. Interhemispheric connectivity may support comprehension or compensation when left-hemisphere pathways are compromised, but its association with production can differ by tract and recovery stage (Sihvonen et al., 2023). Within-hemisphere structural integrity constrains information transfer along dorsal and ventral language pathways (Wilmskoetter et al., 2022), while resting-state functional connectivity and network modularity may index the capacity for coordinated recruitment and have been associated with narrative recovery and therapy responsiveness (Duncan and Small, 2016; Falconer et al., 2024; Tao and Rapp, 2019). These metrics should therefore be interpreted in relation to the language domain, lesion burden, and longitudinal outcome rather than as a single global index of adaptive reorganization.

Portable measurements provide a practical implementation route within this biomarker layer. fNIRS can characterize task-related HbO/HbR responses, resting-state connectivity, and lateralization in superficial frontal, temporal, and parietal cortex; studies in aphasia have demonstrated feasibility for language and domain-general processing, early post-stroke connectivity, and cognitive-interference paradigms (Gilmore et al., 2021; Meier et al., 2023; Lu et al., 2025). Functional mapping with fNIRS has also been explored for neuromodulation target selection (Hara et al., 2017; Chang et al., 2022), while EEG can quantify the oscillatory and time-resolved dynamics of the selected network.

Modality selection should follow the clinical mechanism: EEG is suited to millisecond-scale acoustic, linguistic, and oscillatory dynamics, whereas fNIRS is useful when bedside tolerance and localized superficial hemodynamic measurement are priorities. Their combined use is most informative when each modality answers a prespecified and non-redundant question.

### 6.4 Clinical decision layer

The fourth layer translates profile and biomarker information into clinical decisions about prognosis, therapy, rehabilitation intensity, neuromodulation targets, and monitoring. These measures are intended to complement—not replace—severity, lesion characteristics, time post-stroke, clinician judgment, and patient goals.

For prognosis prediction, biomarkers may help identify which patients are likely to recover spontaneously, respond to therapy, or require long-term compensatory strategies. Early functional activation has been shown to predict language outcome after stroke (Saur et al., 2006), and longitudinal imaging studies have demonstrated dynamic phases of language-network reorganization during recovery (Stockert et al., 2020). Structural connectivity modeling has also been used to predict post-stroke language function from routinely acquired clinical MRI data (Hildesheim et al., 2024; Tao and Rapp, 2019), while resting-state functional connectivity may predict responsiveness to language therapy in chronic post-stroke aphasia (Falconer et al., 2024). Together, these findings suggest that brain-network measures could complement behavioral severity scores in estimating recovery potential.

For therapy selection, the profile can generate testable hypotheses: lexical-semantic difficulty may motivate lexical-semantic approaches; grammatical difficulty may motivate sentence-level treatment; discourse difficulty may support narrative intervention; executive-predictive limitations may require monitoring and flexible response selection; and functional communication limitations may require partner or participation-oriented strategies. Treatment studies combining behavioral rehabilitation with imaging measures provide additional context for testing imaging-informed therapy selection and response (Meinzer et al., 2012; Fleming et al., 2020).

For neuromodulation target selection, network biomarkers may be particularly useful. Non-invasive brain stimulation has been used to modulate left perilesional regions, right-hemisphere homologues, or other language-related regions, but optimal targets likely differ across patients. fNIRS-guided selective rTMS has shown that functional activation patterns can guide stimulation targets in post-stroke aphasia (Hara et al., 2017), and fNIRS-guided high-frequency rTMS over activated speech-related areas combined with speech therapy has been associated with improved language function and altered network connectivity (Chang et al., 2022). A multimodal strategy could extend this approach for stimulation methods such as transcranial alternating current stimulation: fNIRS could identify the regional cortical target during a relevant language task, while EEG could characterize the frequency, phase, and timing of oscillatory activity to be targeted. This proposal remains to be tested prospectively, including whether multimodal targeting improves outcomes beyond anatomy- or activation-guided selection alone.

For rehabilitation stratification, patients can be described by combinations of behavioral and network features rather than fixed biomarker-defined types. Whether these combinations improve treatment matching beyond conventional clinical variables remains a prospective empirical question.

### 6.5 Outcome monitoring layer

The fifth layer focuses on longitudinal monitoring of both language behavior and the neural systems that support it. Conventional outcomes remain necessary but should be complemented by connected-speech metrics, participation measures, functional communication outcomes, and selected biomarkers of network change.

Connected-speech monitoring can include informativeness, lexical diversity, content-word production, grammatical complexity, fluency, coherence, and communicative efficiency. Participation measures test whether gains transfer to daily life, which is ultimately more important than improvement on isolated test items alone.

Biomarker monitoring can provide additional information about mechanisms of change. Portable EEG or fNIRS can be repeated during rehabilitation to track selected electrophysiological or hemodynamic measures, while fMRI can characterize treatment-induced reorganization and language-domain-specific plasticity (Barbieri et al., 2023). Structural and functional connectivity measures can test whether treatment is accompanied by network normalization, compensatory reorganization, or altered interhemispheric coupling.

Monitoring also creates a feedback loop. Behavioral improvement without measurable network change may reflect compensation or biomarker insensitivity; neural change without functional improvement may signal limited transfer. Concordant improvement provides stronger evidence that rehabilitation is modifying systems relevant to real-world communication.

In summary, the framework advances only through reproducible links among communication targets, tasks, multivariate profiles, neural measures, clinical hypotheses, and longitudinal outcomes. It should be revised if profiles are unstable, neural measures add no value beyond conventional variables, or biomarker-informed decisions fail to improve patient-centered communication.

## 7 Discussion: methodological challenges and future directions

Several methodological challenges must be addressed before ecological language biomarkers can be used routinely, including task standardization, measurement heterogeneity, high-dimensional feature selection, neurophysiological artifacts, cross-language validity, and prospective clinical validation.

### 7.1 Standardization of naturalistic tasks

A major challenge is the lack of standardized naturalistic language tasks. Connected speech, narrative production, picture description, story retelling, conversation, and functional communication samples are all used to approximate real-world language use, but they differ substantially in elicitation procedures, task demands, scoring systems, and clinical interpretation (Stark et al., 2021b; Cordella et al., 2024). For example, picture description tasks impose visual and semantic constraints, whereas personal narratives and conversational exchanges place greater demands on autobiographical memory, pragmatic adaptation, topic maintenance, and executive control (Alyahya et al., 2020; Casilio et al., 2025; Barberis et al., 2026). Therefore, findings from one naturalistic task cannot be assumed to generalize directly to another.

Future studies should distinguish clearly among task types rather than treating all discourse samples as equivalent. At minimum, studies should report the elicitation method, stimulus type, task duration, examiner prompts, transcription procedures, coding rules, and whether the task primarily assesses production, comprehension, interaction, or functional communication (Stark et al., 2021b; Cordella et al., 2024). Standardized reporting would improve reproducibility and allow evidence synthesis across studies. A tiered task battery may be particularly useful: one brief connected speech task for clinical feasibility, one narrative or discourse task for richer neuropsychological phenotyping, and one interactive communication task for ecological validity.

Standardization should not mean eliminating ecological richness. Instead, the goal should be controlled naturalism: tasks should be sufficiently structured to allow comparison across patients and studies, but sufficiently naturalistic to capture real-world communication processes. This balance is especially important for neuroimaging studies, where task design affects both behavioral performance and neural activation patterns (Gilmore et al., 2021; Maloney et al., 2023; De Clercq et al., 2025). Future ecological fNIRS and EEG paradigms should therefore specify the linguistic, cognitive, and motor demands of each task and align these demands with predefined neuropsychological constructs.

### 7.2 Measurement heterogeneity in connected speech

A second challenge is measurement heterogeneity. Connected speech studies use a wide range of metrics, including word count, speech rate, mean length of utterance, content information units, lexical diversity, lexical sophistication, syntactic complexity, fluency, informativeness, coherence, error types, paraphasias, pauses, and communicative efficiency (Stark et al., 2021b; Cordella et al., 2024). Although this multidimensionality is valuable, it creates difficulty for comparing studies, conducting meta-analyses, and developing clinically interpretable benchmarks.

This problem is not unique to naturalistic language assessment. Aphasia treatment research more broadly has long suffered from outcome heterogeneity, motivating the development of core outcome sets such as the Research Outcome Measurement in Aphasia consensus statement (Wallace et al., 2019). The ROMA initiative identified essential outcome constructs including language, communication, quality of life, emotional wellbeing, and patient-reported satisfaction or impact of treatment (Wallace et al., 2019; Wallace et al., 2023). Naturalistic language assessment should align with this broader movement toward standardized but meaningful outcome measurement.

A practical future direction is to organize connected speech metrics into a limited number of neuropsychological domains. For example, lexical measures can index lexical retrieval; informativeness and coherence can index semantic integration and discourse planning; syntactic complexity can index grammatical organization; pause patterns and speech rate can index fluency and speech-motor output; and repair behavior or topic maintenance can index executive-linguistic control (Wilmskoetter et al., 2019; Gleichgerrcht et al., 2021; Ding et al., 2023; Cordella et al., 2024; Casilio et al., 2025). Such domain-based organization would allow different studies to use different tools while still mapping outcomes onto comparable constructs.

The field also needs consensus on minimal reporting standards. Studies should report transcription reliability, coding reliability, sampling duration, task type, language context, severity distribution, and whether metrics are manually coded, automatically extracted, or derived from natural language processing pipelines (Cordella et al., 2024). Automated speech and language analysis may improve scalability, but derived metrics still require validation against clinician-coded measures and across aphasia severity levels, languages, and discourse contexts. Without such validation, automated metrics risk becoming technically impressive but clinically opaque.

### 7.3 The high-dimensional feature space of naturalistic language

Naturalistic language creates a high-dimensional analytic space: acoustic, phonological, lexical, surprisal, grammatical, semantic, discourse, pragmatic, and interactional features may be correlated, while neuroimaging adds choices of temporal lags, regions, frequency bands, connectivity metrics, and regularization. Without prespecification, this flexibility increases researcher degrees of freedom and overfitting risk.

Recent work illustrates both the opportunity and the problem. Shain et al. compared multiple computational characterizations of working-memory demand during naturalistic comprehension (Shain et al., 2022); Kries et al. modeled acoustic and linguistic representations during continuous speech in post-stroke aphasia (Kries et al., 2024); and Rafferty et al. showed that two syntactic predictors can yield distinct neural timing effects related to comprehension (Rafferty et al., 2026). Future studies should preregister a limited feature set or a hierarchical analysis plan, report collinearity and dimensionality-reduction decisions, and validate latent dimensions out of sample. The goal is not to extract every available feature, but to identify a small set that is reliable, interpretable, and incrementally useful.

### 7.4 Measurement validity and modality-specific limitations of portable EEG and fNIRS

The modalities also differ in biological and anatomical interpretation. EEG directly measures electrical activity with high temporal resolution but has limited source-localization precision, particularly in brains distorted by stroke lesions. fNIRS measures the hemodynamic consequence of neural activity and provides localized sampling of superficial cortex, but it has coarser temporal resolution, limited depth and whole-brain coverage, and sensitivity to neurovascular and extracerebral signals. Neither modality should therefore be treated as a stand-alone aphasia biomarker.

Quality control should be built into acquisition and analysis. EEG studies should report electrode placement, impedance or signal-quality criteria, artifact detection and component-removal procedures, reference scheme, trial retention, frequency-band or tracking-model specification, and test–retest reliability. fNIRS studies should report source-detector geometry, short-separation channels where available, motion detection and correction, systemic physiology monitoring, HbO/HbR modeling, channel rejection, and sensitivity analyses. For speech protocols, correction methods should be selected and justified rather than relying only on trial rejection; wavelet-based correction and speech-specific processing strategies are among the approaches that have improved fNIRS data quality (Brigadoi et al., 2014; Novi et al., 2020).

Lesion-aware localization remains desirable for both modalities but is more straightforward to recommend than to implement in current stroke studies. Individual electrode or optode digitization and structural MRI co-registration may improve anatomical interpretation when available; however, standardized and widely accessible workflows for lesion-aware fNIRS localization remain limited. Lesion cavities, atrophy, and tissue displacement can make canonical scalp-to-cortex mappings unreliable, so studies should report registration constraints explicitly. Most importantly, preprocessing decisions should be prespecified, and candidate measures should demonstrate test–retest reliability and validation against conventional language scores, naturalistic behavior, and functional communication outcomes before clinical interpretation.

### 7.5 Cross-language and cross-cultural validation

Most naturalistic language studies in aphasia have been conducted in a limited number of languages, with English disproportionately represented (Cordella et al., 2024). This creates a major challenge because connected speech, discourse structure, syntax, lexical diversity, informativeness, and narrative organization are language dependent. Metrics developed in English may not transfer directly to languages with different morphology, word order, discourse conventions, writing systems, or pragmatic norms.

Cross-language and multilingual aphasia research shows that language proficiency, linguistic distance, premorbid language use, and treatment language can influence recovery and generalization (Kohnert, 2009; Conner et al., 2018; Goral et al., 2023). Kohnert emphasized that treatment effects in one language may or may not generalize to another language in bilingual aphasia, depending on patient and language factors (Kohnert, 2009). More recent evidence suggests that cross-language generalization can occur, but its magnitude and direction depend on language proficiency, similarity, and therapy targets (Conner et al., 2018; Goral et al., 2023). These findings are directly relevant to naturalistic assessment because discourse and functional communication are shaped by language-specific and culture-specific expectations.

For future research, ecological aphasia assessment should be developed with cross-linguistic validity in mind from the beginning. This means that studies should report participants’ language background, premorbid proficiency, dominant language, literacy level, dialect, and sociocultural communication context (Kohnert, 2009; Conner et al., 2018; Goral et al., 2023). It also means that connected speech metrics should be adapted rather than translated mechanically. For example, lexical diversity, syntactic complexity, and discourse coherence may require language-specific operational definitions.

Cross-cultural validation is equally important. Functional communication depends not only on linguistic ability but also on social roles, family communication patterns, rehabilitation goals, and environmental support (Wallace et al., 2019; Schumacher et al., 2020). A task that is ecologically valid in one cultural setting may not be equally meaningful in another. Future studies should therefore include culturally relevant discourse prompts, patient-centered communication outcomes, and participation-based measures that reflect daily communication demands across settings.

### 7.6 Longitudinal prediction and clinical implementation

The final challenge is clinical translation. Many existing studies demonstrate associations between naturalistic language measures, lesion characteristics, connectivity, or activation patterns, but fewer studies test whether these measures improve prognosis prediction, treatment selection, or outcome monitoring beyond conventional clinical variables (Saur et al., 2006; Stockert et al., 2020; Iorga et al., 2021; Kristinsson et al., 2022; Falconer et al., 2024). For ecological biomarkers to become clinically useful, they must demonstrate incremental value: they should explain or predict outcomes better than aphasia severity, lesion size, time post-stroke, age, and baseline language scores alone.

Longitudinal studies are particularly important because aphasia recovery is dynamic. Imaging studies have shown that language networks reorganize over time after stroke, with changes in left-hemisphere activity, right-hemisphere recruitment, and connectivity patterns across recovery phases (Saur et al., 2006; Stockert et al., 2020). Behavioral and connectivity studies further suggest that baseline functional connectivity and patient-level factors may predict therapy response in chronic aphasia (Iorga et al., 2021; Kristinsson et al., 2022; Falconer et al., 2024). These findings support the possibility of individualized prediction, but more work is needed to integrate naturalistic language phenotypes with longitudinal biomarkers.

Clinical implementation should proceed in stages: standardize naturalistic tasks, establish reliable behavioral metrics, validate imaging biomarkers for feasibility and sensitivity to change, build multimodal prediction models, and finally test prospectively whether biomarker-informed decisions improve outcomes.

A useful middle ground is to relate neural responses from controlled tasks to naturalistic communication outcomes and, conversely, compare naturalistic neural measures with constrained behavioral constructs. Concordance supports cross-context generalization; dissociation may reveal limited ecological transfer or additional interacting demands. These comparisons should be designed prospectively.

Clinical feasibility must remain central. A precision framework that requires long scanning sessions, complex postprocessing, or costly multimodal imaging may have limited value in routine rehabilitation. By contrast, brief naturalistic language sampling combined with portable fNIRS or EEG may offer a more scalable pathway, particularly if automated speech analysis and standardized preprocessing pipelines become available (Novi et al., 2020; Gilmore et al., 2021; Cordella et al., 2024; De Clercq et al., 2025). Ultimately, ecological biomarkers should help clinicians answer practical questions: What type of language difficulty is most limiting this patient’s communication? Which neural systems appear preserved or compensatory? Which therapy target is most appropriate? Is the patient improving in ways that transfer to daily life?

### 7.7 Limitations of this review

This review is an integrative synthesis supported by a structured search and descriptive thematic map rather than an exhaustive systematic review or formal evidence-grading exercise. Targeted searches were added for cortical tracking, phonology, grammar, and predictive processing. Eighty-three records characterized thematic breadth; 32 prioritized full texts were reviewed, 27 were retained as core/supporting evidence, and five were excluded from the core synthesis. Fourteen potentially relevant reports remained unavailable in full. Heterogeneity in tasks, stroke stages, metrics, and analytic pipelines still limits direct comparison.

## 8 Conclusion

Naturalistic language assessment complements constrained aphasia testing by quantifying several linguistic and cognitive sources of difficulty within the same extended communicative context and by revealing how those sources interact during language use.

The available evidence supports a multivariate account rather than a set of discrete linguistic phenotypes. Lexical-semantic, phonological, grammatical, discourse, executive-predictive, and speech-motor dimensions overlap, and their neural correlates vary with lesion anatomy, residual network integrity, task demands, and recovery stage. Naturalistic behavior therefore provides a rich measurement context for testing brain-behavior relationships without assuming one-to-one mappings.

We propose an operational framework in which a defined communication target is sampled with an appropriate task, converted into a reproducible multivariate feature profile, compared with lesion and neural measures, and validated against longitudinal or treatment-related outcomes. Clinical value depends on whether neural information adds reproducible information beyond conventional severity measures and whether biomarker-informed decisions ultimately improve communication outcomes.

Future research should develop standardized, ecologically meaningful, and biomarker-informed assessment pathways. Priorities include harmonized naturalistic language tasks, reliable connected-speech metrics, artifact-aware EEG/fNIRS protocols, cross-language validation, and longitudinal studies that test whether ecological biomarkers improve clinical decision-making and patient-centered communication outcomes.
