# Supplementary material for: Naturalistic language assessment and brain-network biomarkers in post-stroke aphasia for precision neurorehabilitation
Source: Front Hum Neurosci. 2026 Oct 15;20:1966552. doi: 10.3389/fnhum.2026.1966552 (PMC13717245; doi:10.3389/fnhum.2026.1966552)
Supplement: Supplementary file 1 [file Supplementary_file_1.docx]

Supplementary Table S1. Full-text records retained as core or supporting evidence for detailed synthesis (n = 27).

| **Full-text record (first author, year)** | **Primary evidence domain** | **Role in detailed synthesis** |
| --- | --- | --- |
| Efficacy of spoken word comprehension therapy in chronic aphasia: cross-over trial with structural imaging (Fleming et al., 2020) | Treatment response / structural imaging | Supporting evidence for imaging-informed treatment response |
| Language Mapping in Aphasia (Wilson et al., 2019) | Language mapping / task fMRI | Core methodological support for individualized language mapping |
| The neural architecture of the language comprehension network: lesion and connectivity analyses (Turken and Dronkers, 2011) | Comprehension network / lesion-connectivity | Core support for distributed language-comprehension networks |
| The effects of lesion and treatment-related recovery on functional network modularity in post-stroke dysgraphia (Tao and Rapp, 2019) | Network modularity / treatment-related recovery | Supporting network-level recovery evidence |
| Independent contributions of structural and functional connectivity: Evidence from a stroke model (Keator et al., 2021) | Structural and functional connectivity | Core support for complementary connectivity information |
| The first decade of research on constraint-induced treatment approaches for aphasia rehabilitation (Meinzer et al., 2012) | Rehabilitation / functional communication | Supporting rehabilitation context |
| Automated Tractography for the Assessment of Aphasia in Acute Care Stroke Rehabilitation: A Case Series (Mochizuki et al., 2023) | Tractography / acute-care assessment | Supporting clinical translation of structural connectivity |
| Language Recovery after Brain Injury: A Structural Network Control Theory Study (Wilmskoetter et al., 2022) | Structural network control / recovery | Core support for network-level recovery models |
| Discourse abilities in post-stroke aphasia: macro- and micro-structural discourse properties and white matter correlates (Jacobs et al., 2026) | Discourse / white matter | Core naturalistic discourse-neuroanatomy evidence |
| Longitudinal prediction of naming and connected speech in post-stroke aphasia: acute white matter characteristics (Desjardins et al., 2026) | Connected speech / longitudinal prediction | Core prognosis and precision-rehabilitation evidence |
| Right hemispheric structural connectivity and poststroke language recovery (Sihvonen et al., 2023) | Right-hemisphere structural connectivity | Core evidence for conditional right-hemisphere contributions |
| Functional MRI Activation During Unscripted Discourse in People With Poststroke Aphasia (Maloney et al., 2023) | Unscripted discourse / fMRI | Core naturalistic task-fMRI evidence |
| A unified model of post-stroke language deficits including discourse production and their neural correlates (Alyahya et al., 2020) | Discourse / multivariate language model | Core conceptual and empirical anchor |
| Right hemisphere grey matter structure and language outcomes in chronic left hemisphere stroke (Xing et al., 2016) | Right-hemisphere grey matter | Core evidence on direction and domain of right-hemisphere associations |
| Domain-general subregions of the medial prefrontal cortex contribute to recovery of language after stroke (Geranmayeh et al., 2017) | Domain-general control network | Core support for executive/control-network contributions |
| Increased Modularity of Resting State Networks Supports Improved Narrative Production in Aphasia Recovery (Duncan and Small, 2016) | Narrative / resting-state networks | Core evidence linking narrative recovery to network organization |
| The verbal, non-verbal and structural bases of functional communication abilities in aphasia (Schumacher et al., 2020) | Functional communication | Core ecological/functional communication evidence |
| Impaired discourse content in aphasia is associated with frontal white matter damage (Ding et al., 2023) | Discourse content / white matter | Core link between discourse informativeness and frontal white matter |
| Partial least squares multimodal analysis of brain network correlates of language deficits in aphasia (Kristinsson et al., 2025) | Multimodal brain networks | Core support for multimodal network integration |
| The lesion correlates of impaired content word fluency during spoken discourse in aphasia (Alyahya et al., 2026) | Spoken discourse / lesion mapping | Core evidence for lesion correlates of connected-speech features |
| Functional Network Changes After High-Frequency rTMS Over the Most Activated Speech-Related Area Combined With Speech Therapy (Chang et al., 2022) | Neuromodulation / functional networks | Supporting evidence for biomarker-informed target selection |
| Neuroanatomical structures supporting lexical diversity, sophistication, and phonological word features during discourse (Wilmskoetter et al., 2019) | Discourse linguistic features / neuroanatomy | Core evidence for multilevel lexical/phonological features |
| Discourse- and lesion-based aphasia quotient estimation using machine learning (Riccardi et al., 2024) | Discourse / lesion / machine learning | Core evidence for multivariate prediction and translation |
| Separate neural systems support representations for actions and objects during narrative speech in post-stroke aphasia (Gleichgerrcht et al., 2016) | Narrative semantics | Core evidence for semantic organization during narrative speech |
| Neural bases of elements of syntax during speech production in patients with aphasia (Gleichgerrcht et al., 2021) | Grammar / lesion-symptom mapping | Core evidence for grammatical dimensions in speech production |
| Treatment-induced neural reorganization in aphasia is language-domain specific: evidence from a large-scale fMRI study (Barbieri et al., 2023) | Treatment-induced reorganization / fMRI | Core longitudinal treatment-plasticity evidence |
| Lesion correlates of conversational speech production deficits (Borovsky et al., 2007) | Conversation / lesion mapping | Core evidence for lesion correlates of conversational speech |

Note: Additional methodological, theoretical, diagnostic, and context-setting references are cited in the main text but are not counted among the 27 retained full-text records.
